# Ultrahigh Resolution Polarization Sensitive Optical Coherence Tomography of the Human Cornea with Conical Scanning Pattern and Variable Dispersion Compensation

**DOI:** 10.3390/app9204245

**Published:** 2019-10-11

**Authors:** Florian Beer, Rahul P. Patil, Abhijit Sinha-Roy, Bernhard Baumann, Michael Pircher, Christoph K. Hitzenberger

**Affiliations:** 1 Center for Medical Physics and Biomedical Engineering, Medical University of Vienna, 1090 Vienna, Austria; 2 Institute of Applied Physics, Vienna University of Technology, 1040 Vienna, Austria; 3 Narayana Nethralaya Foundation, Bengaluru 560099, India

**Keywords:** cornea, optical coherence tomography, polarization sensitive imaging, dispersion compensation, ultra-high resolution

## Abstract

Noninvasive corneal imaging is essential for the diagnosis and treatment control of various diseases affecting the anterior segment of the eye. This study presents an ultrahigh resolution polarization sensitive optical coherence tomography instrument operating in the 840 nm wavelength band that incorporates a conical scanning design for large field of view imaging of the cornea. As the conical scanning introduces a dispersion mismatch depending on the scanning angle, this study implemented variable, location dependent, numerical dispersion compensation in order to achieve high axial resolution throughout the imaged volume. The corneal images were recorded in vivo in healthy volunteers showing various details of corneal structures.

## 1 Introduction

Human vision starts with image formation on the retina where the light is detected and converted into electrical signals that are preprocessed and then finally sent to the brain. Imaging in the eye is achieved by refraction at the cornea and the lens. Thereby, the total refractive power of the eye is dominated by the cornea because at the surface of this tissue, the refractive index change is higher than at all other interfaces within the eye. Thus, diseases affecting the corneal shape, such as keratoconus, have a large impact on the visual acuity of a subject.

The human cornea consists of various layers that can be anatomically separated, starting with the outermost layer, into the epithelium, Bowman’s layer, stroma, Descemet’s membrane and endothelium [1]. The epithelium represents the barrier between the tissue and outside environment and has a thickness of 40–50 μm or 5 to 6 cell layers [1]. Bowman’s layer appears smooth with a thickness of ~15 μm and helps maintaining the shape of the cornea [1]. The thickest layer of the cornea is the stroma that consists of 200–250 lamellae of 0.2–2.5 μm thickness each consisting of parallel arranged and densely packed collagen fibrils of 25–35 nm thickness [2]. Thus, each lamella consists of 8–100 fibrils. The regular arrangement of these fibrils introduces form birefringence, an optical effect that can be measured using polarization sensitive methods, such as polarimetry [3] or polarization sensitive optical coherence tomography (PS-OCT) [4–6]. Descemet’s membrane, a layer with approximately 10 μm thickness, separates the stroma from the endothelium which appears as a honeycomb like mono cellular layer [1]. All corneal layers in the healthy eye are highly transparent which sets high demands on optical methods investigating the cornea in terms of system sensitivity.

A variety of methods are available for imaging corneal structures and alterations thereof such as ultrasound imaging [7], confocal microscopy [8] and optical coherence tomography (OCT) [9]. Specifically, OCT can be regarded as a routine clinical tool as the technology provides contact free and non-invasively high axial resolution images of the cornea with a large field of view [10,11]. As the shape of the cornea is approximately spherical with an extension of 11 mm (lateral) × 5mm (axial), there are some challenges for OCT imaging, specifically in the case of high axial resolution (ultrahigh resolution) imaging. Outside the central corneal area of 2–3 mm diameter, the probing beam has a large inclination with respect to the corneal surface. As a consequence, a lower OCT signal is detected from these areas because back reflected light is not captured by the system and only a weak backscattered portion of the light remains for detection. In addition, the sharpness of corneal interfaces is lost because of the finite lateral spot size of the imaging beam. Within this spot size, an interface has different axial depth positions. This leads to an axial blurring of the image in this region. Finally, the transverse resolution of such systems is limited by the low numerical aperture of the imaging beam that is needed in order to provide a sufficiently large depth of focus that covers the entire depth and lateral extension of the cornea.

To overcome these issues, we recently developed a conical scanning scheme that achieves nearly perpendicular incidence of the imaging beam on the surface of the cornea [12]. This ensures a high signal quality from the corneal tissue throughout the entire field of view (from corneal limbus to limbus). This initial system was operated with swept source OCT at 1050 nm and achieved an axial resolution of 6.3 μm (in tissue), limited by the sweep range of the used light source. However, highly reproducible and accurate thickness maps of epithelium, Bowman’s layer and stroma in healthy volunteers could be provided with this system [13,14].

This paper extends our conical scanning concept to ultrahigh resolution OCT imaging. To achieve the high axial resolution, the OCT technology was switched to spectrometer-based OCT at 840 nm with an imaging bandwidth of 100 nm that results in a theoretical axial resolution of 4 μm in air. For the conical scanning pattern, an aspheric condenser lens is required that introduces variable dispersion over the scanning field. Thus, we developed an algorithm to compensate for this variable dispersion to achieve high axial resolution throughout the field of view. Similar to our previous instrument, the new system is capable of retrieving polarization sensitive information. Here, this capability is used for investigating corneal structures that are visible in the co-and cross polarized OCT channels, respectively. Thereby, depth varying structures within the corneal stroma are shown that can be observed in the images of the cross polarized detection channel. These may be associated with the depth varying ultrastructure within the organization of the lamellae that are more strictly organized in deeper layers than in anterior layers as has been observed with electron microscopy [1]. The representative image data recorded in healthy volunteers are shown to demonstrate the capabilities of the new instrument.

## 2 Materials and Methods

### 2.1 Experimental Setup

**Figure 1:**
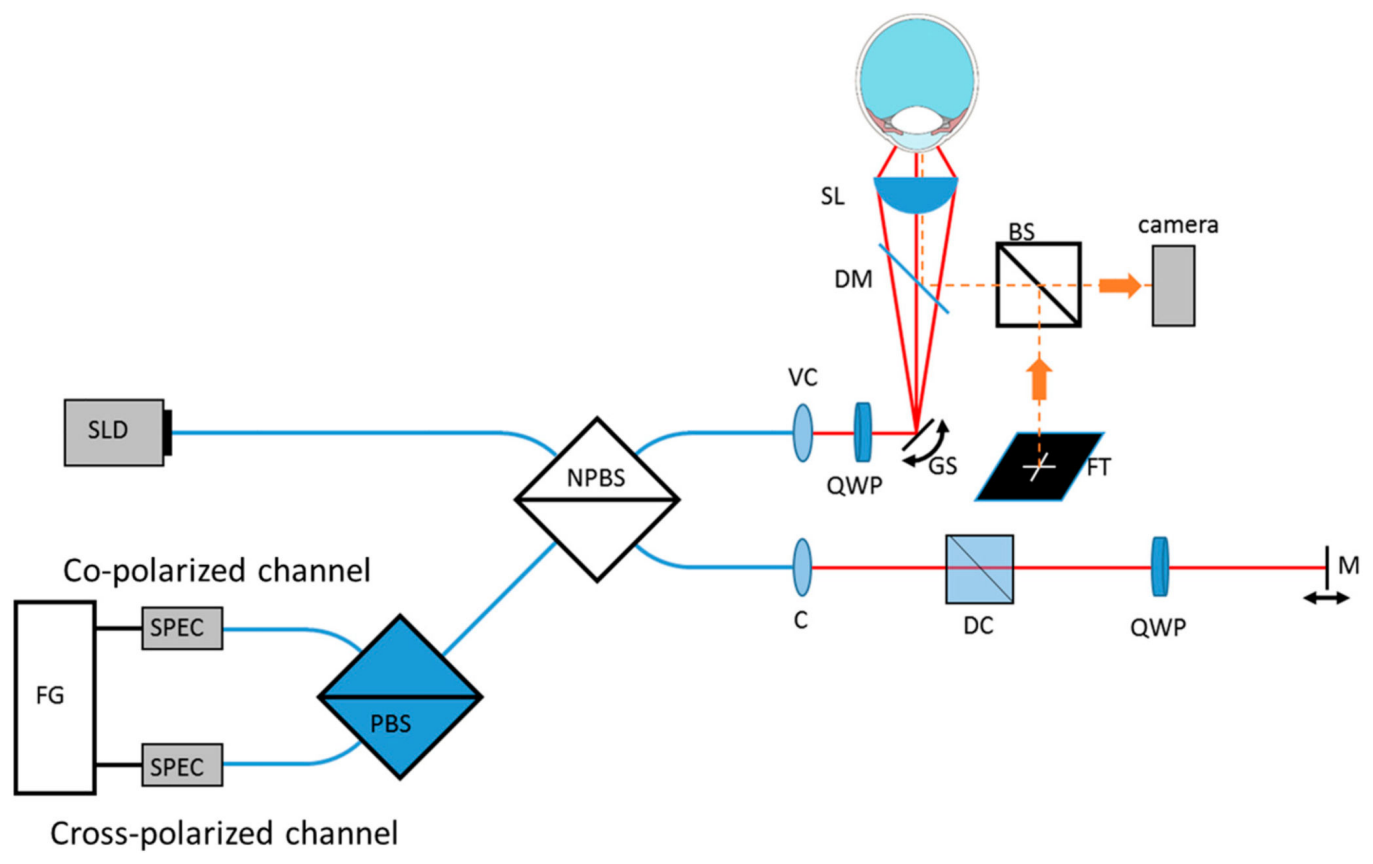
Scheme of the optical setup. SLD (superluminescent diode), NPBS (non-polarizing fiber based beam splitter), VC (variable collimator), QWP (quarter wave plate), GS (galvanometer scanner), DM (dichroic mirror), SL (spherical lens), BS (beam splitter), FT (fixation target), C (collimator), DC (dispersion compensation), M (mirror), PBS (polarizing beam splitter), SPEC (spectrometer), FG (frame grabber). All fibers are polarization maintaining.

The polarization sensitive setup is related to one of our previous systems [15]. A scheme of the system is shown in Figure 1. The light from a superluminescent diode (cBLMD-D-840-HP-I, Superlumdiodes, Cork, Ireland) is sent to a fiber based non-polarizing beam splitter (FOBS-22P, OZ Optics LTD, Ottawa, Ontario, Canada) consisting of polarization maintaining fibers. Further, 80% of the light is directed to the reference arm that includes a dispersion compensation prism pair, a quarter wave plate oriented at 22.5° and a mirror. The light is reflected by the mirror and after double passing the quarter wave plate, the light has a linear polarization state that is oriented at 45° with respect to the incident polarization state. This ensures that equal reference power is provided to both polarization detection channels. Furthermore, 20% of the light is directed to the sample arm where the light exits the fiber and first traverses a variable collimator. With the variable collimator, the focus of the beam can be adjusted to fall within the central depth range of the cornea [12]. Then, the light traverses a quarter wave plate that is oriented at 45°. After passing the wave palate, the light will be in a circular polarization state. The beam is then deflected by two galvanometer scanning mirrors (Thorlabs, Dachau, Germany) and traverses a dichroic mirror and the aspheric condenser lens (Thorlabs ACL5040U-B, Dachau, Germany). The dichroic mirror is used to couple light from the fixation target into the system and to monitor the position of the eye via a camera. A total power of 1 mW is sent to the eye which is well below the corresponding limits for safe exposure given by the laser safety standards [16]. Considering that the light is focused on the cornea, an angular extent of ~49 × 10^−3^ rad on the retina was calculated that results in a safe retinal exposure of up to 19 mW.

The light that is backscattered from the cornea interferes with the light returning from the reference arm at the non-polarizing fiber beam splitter and is split into the two orthogonal polarization states by the fiber based polarizing beam splitter (FOBS-12P, OZ Optics LTD, Ottawa, Ontario, Canada). The light in each polarization state is then spectrally dispersed and detected by two identical spectrometers (CS800-840/114-250-OC2K, Wasatch Photonics, Morrisville, NC, USA). The dispersion compensation prism pair was adjusted with a mirror at the sample location by maximizing the coherence signal (point spread function). The cameras can be operated at different speeds that resulted in A-scan rates of up to 100 kHz. However, in order to increase the sensitivity in the current study, an imaging speed of 50 kHz was used. By attenuating the beam that is incident on a mirror as the sample, a sensitivity of 97 dB for this A-scan rate was measured. The sensitivity drops to 83 dB at 2/3 of the axial measurement range. The measured axial resolution was 4.1μm in air. This corresponds to 2.96 μm in the corneal tissue (assuming a refractive index of 1.385 [17]) which is ~2 times better than our previously reported instrument. The total imaging depth range was 2.9 mm and sufficiently large for imaging the entire cornea because of the used conical scanning pattern.

### 2.2 Imaging Protocol

All measurements adhered to the tenets of the Declaration of Helsinki and were performed under a protocol that was approved by the local ethics committee of the Medical University of Vienna. Informed consent from subjects was obtained prior to the measurements and after possible risks and the nature of the measurements had been explained. For each scanning pattern, this study typically recorded 256 B-scans each consisting of 1024 A-scans. The total recording time for a 3D volume was 5 seconds. Two different scanning patterns were used. In the first scanning pattern, 256 B-scans at the same lateral location (B-M-mode scanning, y-scanner was kept constant) were recorded. In the second scanning pattern, 3D data of the cornea were recorded. The recorded image data covered a field of view of 11 × 11 mm^2^.

### 2.3 Image Post Processing

The interference patterns are recorded in dependence of the respective wavelength and need to be mapped into k (= 2π/wavelength) -space before performing Fourier transformation for retrieving a depth profile (A-scan). However, the exact mapping depends on the alignment of the spectrometer and any differences between the spectrometers influence the polarization sensitive measurements. This can be overcome by either an identical alignment of the spectrometers [18] (which in our case was not possible as we are using commercially available off the shelf spectrometers) or by individual mapping of the interference pattern recorded by each spectrometer into k-space [19]. Although the spectrometers were delivered with calibration factors to associate each spectrometer pixel with the corresponding wavelength, it was found that this conversion was not accurate enough for reliable polarization sensitive imaging. Thus, an auxiliary bulk-optics Michelson interferometer was constructed that was placed into the reference arm of the system while the sample arm was blocked. Both arms of the auxiliary interferometer were terminated by mirrors and the resulting interference fringes were recorded in the two orthogonal polarization channels with both spectrometers. From these fringes, rescaling functions for each channel were derived that allowed the transformation of the spectral data from both spectrometers into the same range in k-space. The resulting calibration functions differed significantly between the channels. This transformation was applied to all spectral data before further post processing and corrected for any mismatch in the alignment of the two spectrometers. In order to remove the DC term and fixed pattern noise, the averaged (mean) spectrum of all A-scans within a B-scan was subtracted from each spectrum.

The dispersion mismatch between the interferometer arms introduces a wavelength dependent phase shift on the spectral data. This can be compensated for in a post processing step introduced by Wojtkowski et al. [20]. The first step was the generation of a complex valued spectrum for each A-scan by applying the Hilbert transformation. Then, we added a quadratic (in wavenumber, for second order (group velocity) dispersion compensation) phase term weighted by a dispersion compensation parameter to the spectrum and performed a Fast Fourier transformation on the real part of the phase corrected data that resulted in an A-scan. In order to find the optimum value for the dispersion compensation parameter, this parameter (200 different values) was varied within a pre-defined and empirically found range (similar to [20]) and calculated the corresponding A-scan. However, instead of a sharpness metric over the entire image as used in [20], the parameter that resulted in the highest peak intensity of each A-scan was used. The value was stored and the procedure was repeated for all A-scans within a B-scan. Thus, the varying dispersion parameters along the B-scan were found. As these resulting parameters fluctuate around the optimum value because of noise issues, the function (dispersion parameter in dependence on A-scan number) was fitted with a polynomial of the 5th order to eliminate this fluctuation. The parameters for each A-scan corresponding to the fit were then used to produce the final image. This procedure was performed independently for all recorded B-scans of the co-polarized channel in order to account for varying dispersion introduced by y-scanning. The same dispersion compensation parameter was then applied to generate the images of the cross-polarized channel. In principle, a third order dispersion parameter can be found in a similar way. However, in our data set, an improvement was not noticed when varying the third order dispersion parameter as the optimum value for this parameter was approximately 0. Thus, the search for this parameter was omitted in our final evaluation procedure.

In a following post processing step, the surface of the cornea was determined via peak detection as described earlier [13] and all A-scans with respect to the found surface were aligned. This step additionally corrected for axial eye motion artifacts that were noticeable because of the rather long measurement time of 5 seconds. Lateral motion was not noticeable in the data sets. Then en-face images were retrieved from the 3D-data set at different depths and further analyzed.

## 3 Results

**Figure 2:**
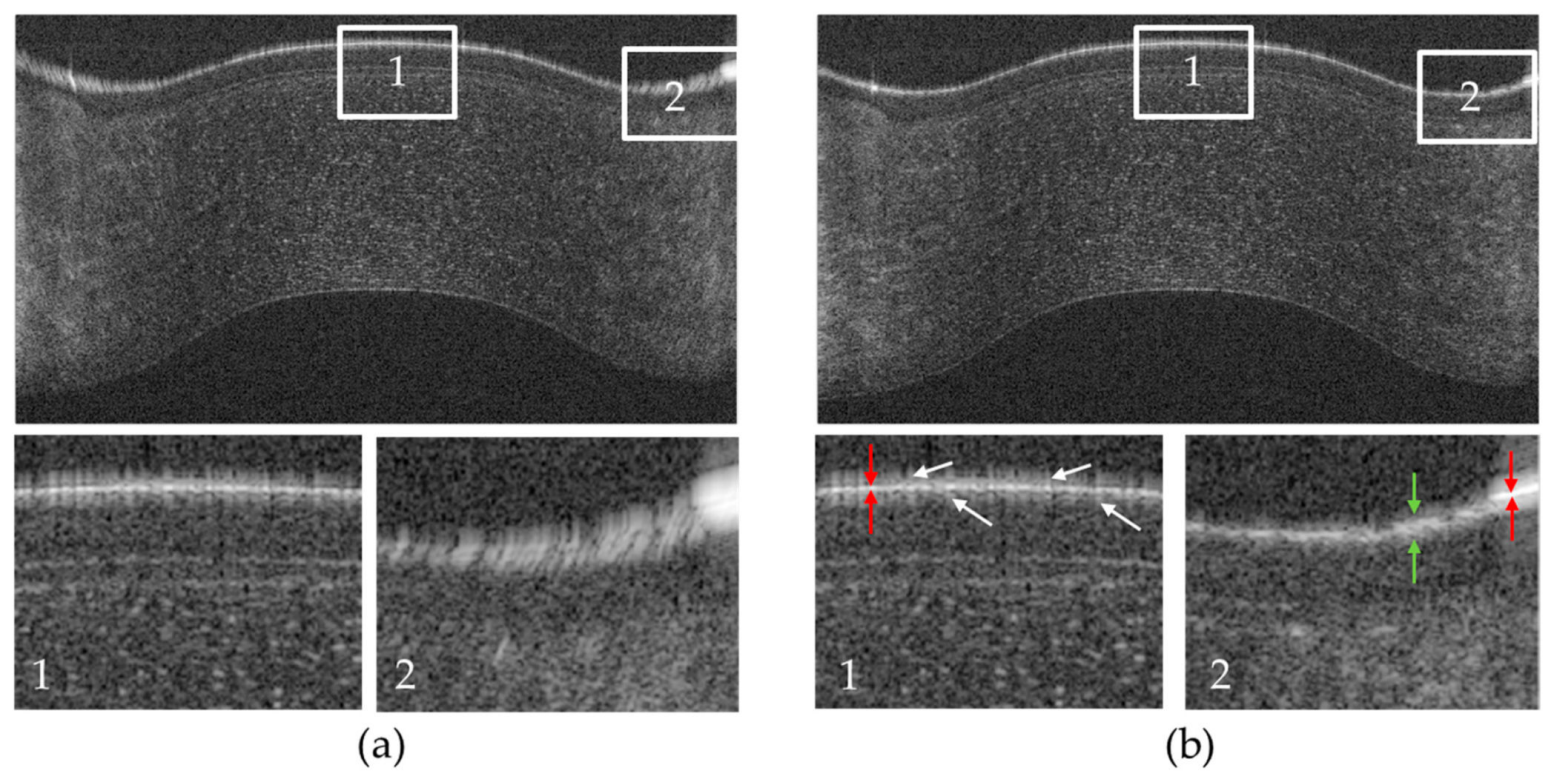
Representative B-scan of the cornea recorded with the instrument in the co-polarized detection channel. (**a**) Without variable dispersion compensation (fixed parameter over the entire B-scan) dispersion is clearly visible at the periphery of the cornea; (**b**) B-scan evaluated with variable dispersion compensation. The lower row shows magnified views (3×) of the regions of interest (1,2) that are marked with white rectangles in the upper row. White arrows point to artefacts at the corneal surface. Red arrows indicate the axial point spread function. Green arrows indicate a thickening of the topmost layer of the epithelium. The field of view of the large images is 11 mm (x) and 867 μm (z).

In order to demonstrate the performance of the variable dispersion compensation, Figure 2 shows a representative B-scan of the cornea recorded in the co-polarized channel prior to the flattening of the image via surface detection without (a) and with (b) variable dispersion compensation. The variable dispersion compensation clearly improves the sharpness of the image in the periphery of the cornea. It should be noted, that in the central part of the cornea, the signal from the surface is very strong. Thus, the side lobes of the coherence function, a result of the spectral shape of the light source, become visible (cf. in Figure 2b the hairy-like appearance of the first surface that is marked with white arrows in the enlarged region of interest 1). The perfect symmetry of these side peaks around the main peak is a good indicator for successful dispersion compensation. Spectral shaping has not been applied in the post processing step. The red arrows in this image mark the extension of the main peak that corresponds to an axial resolution of ~3 μm (~3 pixels) in tissue. Interestingly, the surface appears different in the periphery of the cornea (cf. enlarged region 2). Instead of a single peak, we observed a speckle pattern of high signal that can have a depth extension of up to 12 μm (12 pixels) in tissue that is 4 times larger than the axial resolution of the system. The red arrows in this image mark the coherence function of a reflex that occurs in this region that has the same depth extension as in the central part of the image. Thus, the broadening of the first layer that is observed here is not an artefact caused by residual dispersion but corresponds to an anatomical difference in the periphery of the cornea compared to the central part and might indicate the transition from the corneal to the limbus epithelium. Note that other optical effects can be largely excluded as the endothelium in the region below this thickening still appears as a thin layer.

**Figure 3:**
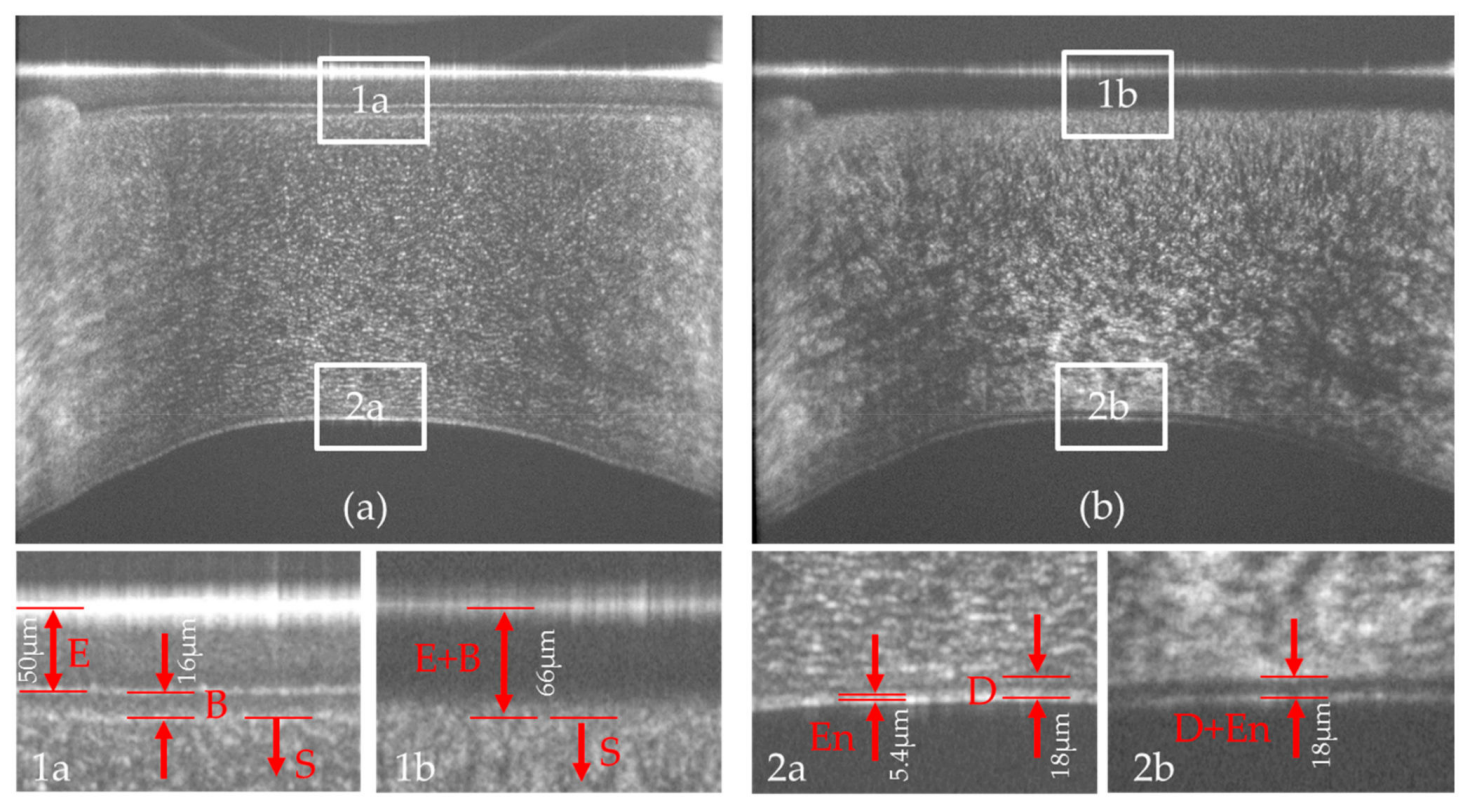
Averaged B-scan (60 times) of the cornea recorded with the instrument in the co- and cross-polarized detection channel. (**a**) Co-polarized detection channel; (**b**) Cross polarized detection channel. The lower row shows magnified views (3×) of the regions of interest that are marked with white rectangles in the upper row. (**1a**) and (**1b**) enlarged view of the anterior part of the cornea, (**2a**) and (**2b**) enlarged view of the posterior part of the cornea of the co- and cross-polarized channel. The field of view of the large images is 11 mm (x) and 860 μm (z). E (epithelium), B (Bowman’s layer), S (stroma), D (Descemet’s membrane), En (endothelfum).

In a next step, 256 B-scans were recorded at the same location. In order to align all B-scans and to flatten the images, the surface of the cornea was detected and all A-scans were aligned according to the segmented surface position. The result for the co- and cross-polarized detection channels is displayed in Figure 3. In the images (cf. also enlarged regions of interest), the individual layers of the cornea can be clearly visualized. However, the contrast in the images differs between the two detection channels. In the co-polarized channel images, the epithelium and Bowman’s layer are nicely separated. A closer look to the enlarged region of interest 1 in Figure 3a reveals that the epithelium can be separated into two regions with slightly varying backscattering properties. The anterior $\frac{3}{4}$ of the layer shows a stronger signal than the remaining quarter of this layer. This might be due to the depth dependent variation of the cellular structure of the epithelium as also observed with (small field of view) ultrahigh resolution OCT and histology [21]. The stroma appears as a rather homogenous structure, densely packed with small and highly reflecting dots, possibly reflections from collagen lamellae or keratocytes. The separation of Descemet’s membrane from the stroma is not well visible in this image. The endothelium appears as a double, highly reflecting interface which is an observation that is similar to previous reports using a small field of view [21,22].

The image retrieved from the cross-polarized channel shows different structures. The epithelium does not alter the polarization state of the light. Thus, only the surface of the cornea (due to the very strong signal from this layer and corresponding cross-talk into this polarization channel) can be observed. The interface between epithelium and Bowman’s layer, although clearly visible in the co-polarized channel, cannot be seen. A signal can be observed in this detection channel starting roughly at the center of Bowman’s layer and continues throughout the stroma. This originates from the depolarization of backscattered light that already originates at Bowman’s layer.

The stroma shows very fine structures in the anterior part that evolve into larger patterns with depth. This gradual change may be associated with the varying collagen density within the stroma. Interestingly, Descemet’s membrane appears very dark in the image indicating that almost no light returning from this layer is in a cross polarized state. Thus, this layer can be well separated from other corneal layers and corresponding layer segmentation may be facilitated by the usage of these images. Another interesting detail is the observation of only a single layer at the endothelium. The double layer structure can only be observed in the image retrieved from the co-polarized channel. It is highlighted here that the double layer is not an artifact caused by side lobes of the coherence function as these appear symmetrically around the main peak and have lower signal strength. The combined image of both channels (total intensity) shows lower contrast of all features presented above.

**Figure 4:**
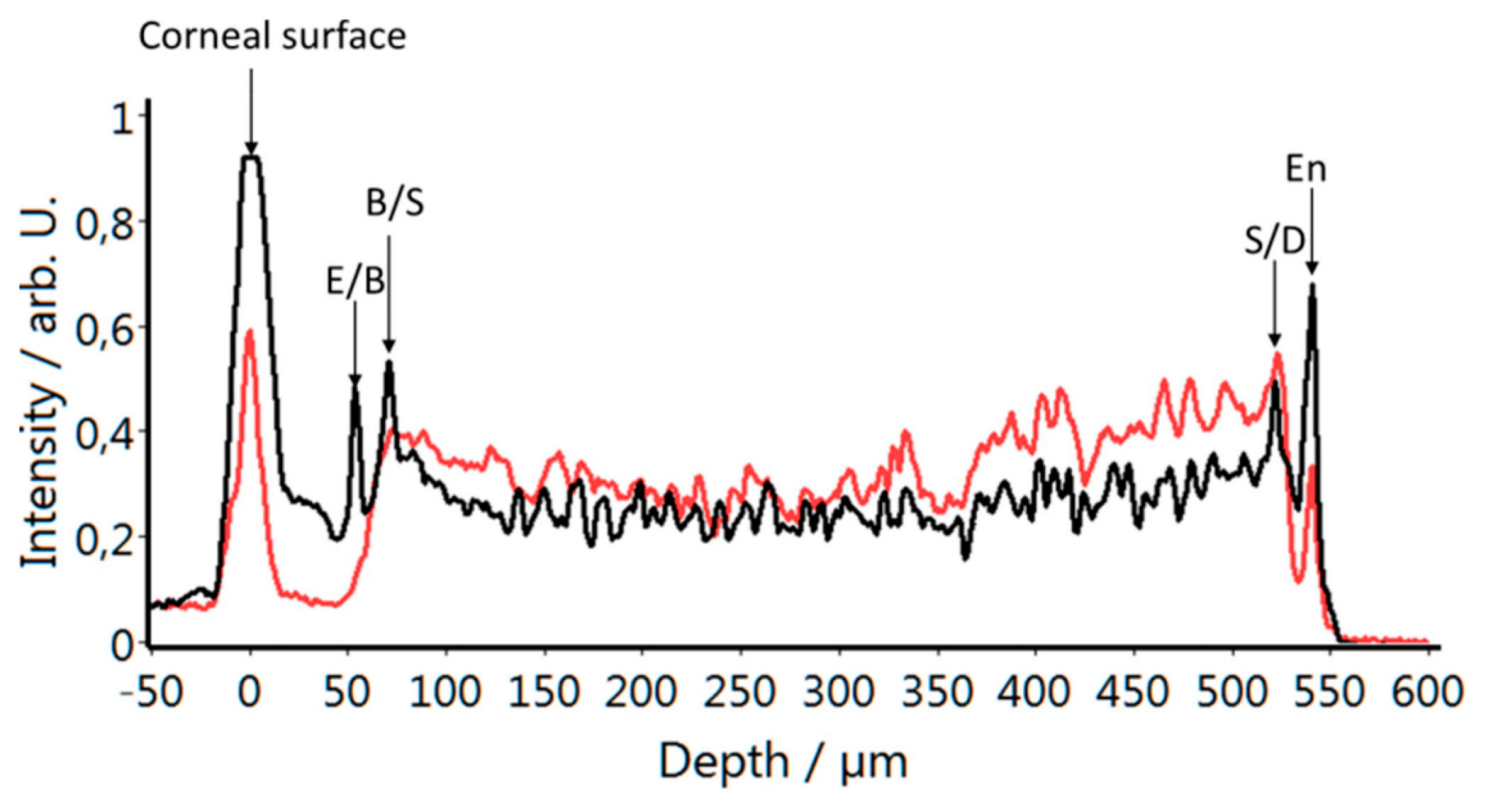
Averaged depth profiles of the central 100 A-scans of the images displayed in Figure 3. Black indicates the co-polarized, red the cross-polarized detection channel. The arrows point to the different corneal interfaces. E (epithelium), B (Bowman’s layer), S (stroma), D (Descemet’s membrane), En (endothelium).

In order to better visualize the signal behavior with the depth in the two polarization channels, on average (mean) of the central 100 A-scans of the images displayed in Figure 3 was plotted. As can be seen in Figure 4, the individual corneal interfaces are clearly visible in the data of the co-polarized detection channel (black curve). The data of the cross polarized detection channel reveals an increase of signal intensity starting at the interface between the epithelium and Bowman’s layer that reaches a maximum at the interface between Bowman’s layer and the stroma.

**Figure 5:**
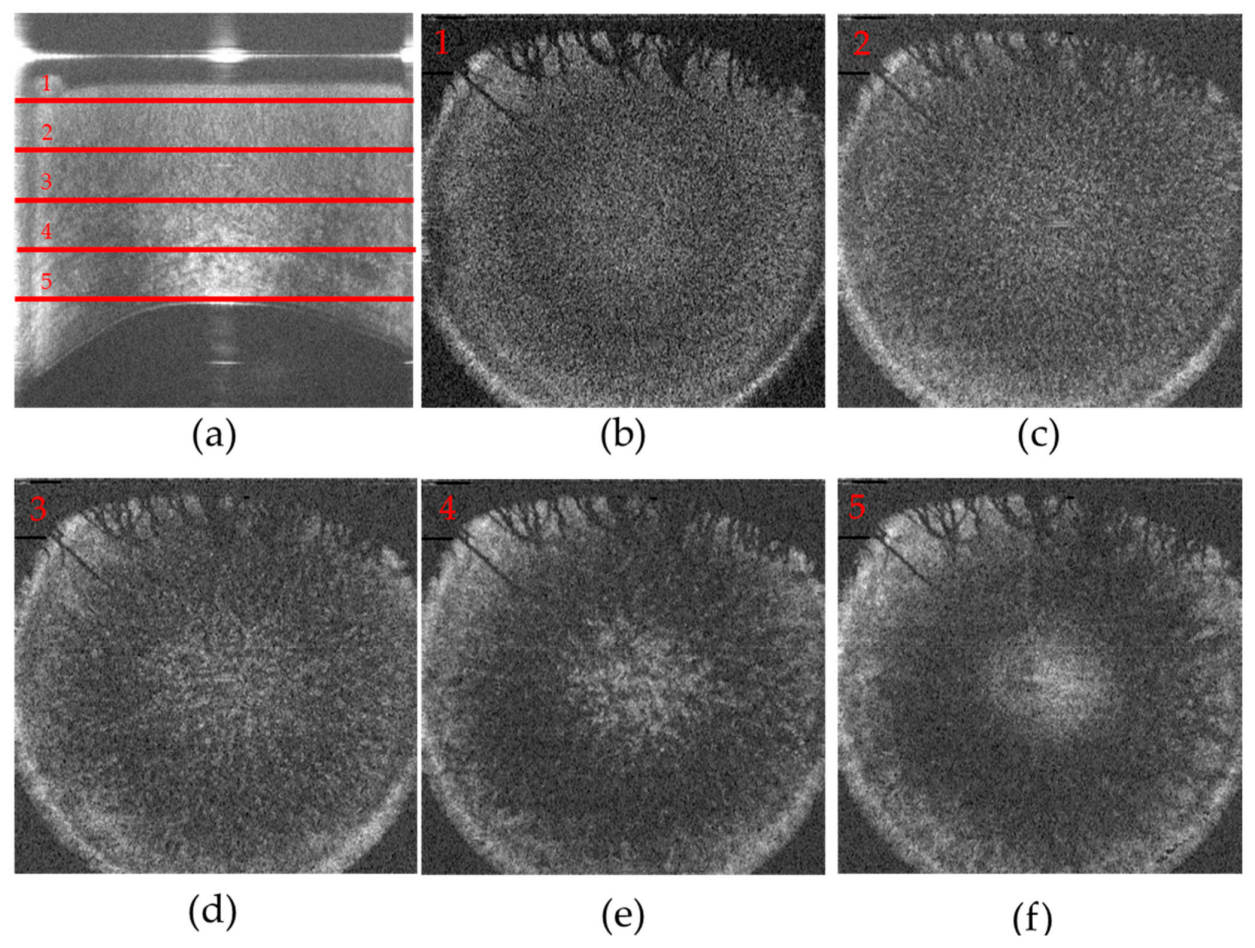
Volume data of the cornea recorded in the cross-polarized detection channel. (**a**) Representative B-scan (lateral average of 10 frames) The depth location of the corresponding en-face images is marked with red horizontal lines; (**b**)–(**f**) En-face frames retrieved at a depth of 100, 200, 300, 400, 500 pixels (101, 202, 303, 404, 505 μm) from the corneal surface (cf. red lines 1–5 in (a); The field of view of (a) is 11 mm (x) and 860 μm (z), of Figure 5b–f is 11 mm (x) and 11 mm (y).

In a next step, 3-dimensional image data were recorded and the depth varying structure of the stroma that is visible in the images of the cross polarized channel was investigated. Figure 5 shows the representative image data. The en-face images retrieved at different imaging depths clearly show that the fine structures that are observed in the anterior part of the stroma evolve into larger connected patterns in deeper regions of the cornea.

**Figure 6:**
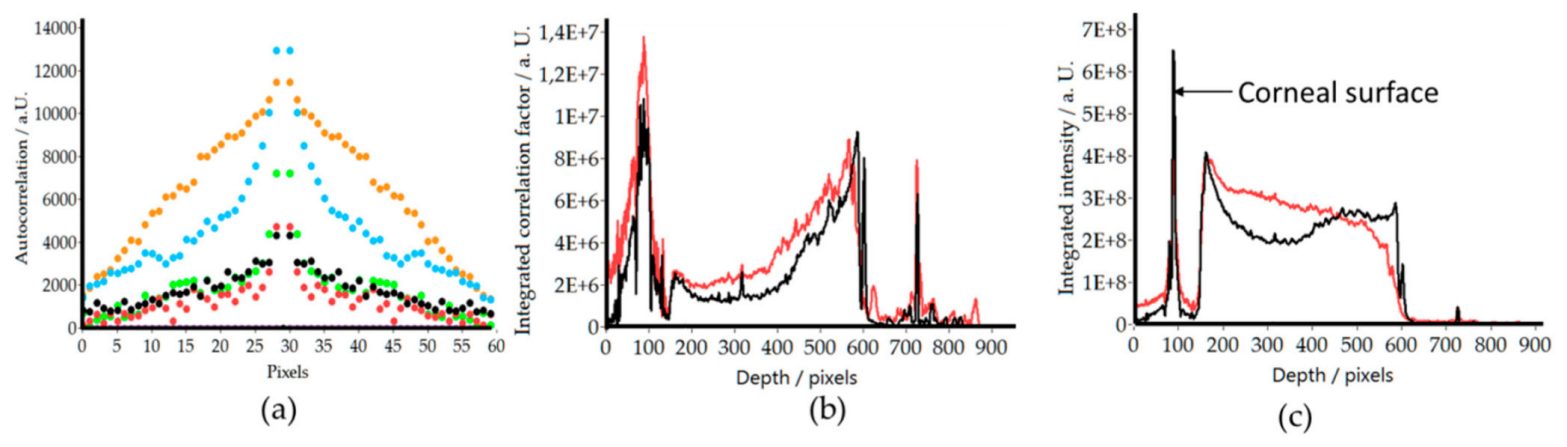
Quantitative evaluation of stromal structure observed in the cross polarized channel. (**a**) Central horizontal autocorrelation function retrieved from en-face images. Black, red, green, blue and orange correspond to an imaging depth of 100, 200, 300, 400, 500 pixels (101, 202, 303, 404, 505 μm) from the corneal surface, respectively. Purple corresponds to the autocorrelation function of residual noise (800 pixel depth); (**b**) Integrated autocorrelation coefficient (IACF) in dependence of imaging depth (the corneal surface is located at pixel 90); (**c**) Integrated intensity in dependence of imaging depth. Black subject 1, red subject 2.

In order to quantify the change of these structures, this study calculated in the central area of the cornea (circular area with 4.2 mm in diameter) the normalized 2D autocorrelation of each image after applying an intensity threshold (in order to minimize the influence of noise all values below this threshold were set to zero). The threshold was set empirically and resulted only in a few visible pixels (due to noise) in areas where no signal was expected. The central point of the resulting autocorrelation function is eliminated (this part was set to zero) to avoid this exceedingly high signal. Figure 6a shows the central part (60 pixels) of the cross section (x-cross_corelation_coefficient plane) of the autocorrelation function. The different colors indicate the varying imaging depths and clearly show first a slow decrease in the total signal followed by an increase of the width of the autocorrelation function with depth. The signal decrease probably results from a decrease of the density of the fine structures that are visible in the anterior part of the stroma (cf. Figure 3b). The width increase can be explained by an enlargement of structures that cause the correlation signal, as can be observed in the corresponding en-face images (cf. Figure 5b–f). For further quantification, the autocorrelation function was integrated over the central part (the extension can be seen in Figure 6a, 1 pixel corresponds to 11 μm) and the result was plotted (integrated auto correlation factor (IACF) over the en-face image) as a function of depth for two healthy subjects (cf. Figure 6b). The slight decrease that is followed by an increase of the IACF with depth is clearly visible in both subjects. This observation might be caused by the varying ultrastructure of the stroma as the fibrils in deeper layers are more strictly organized than in the superficial ones [1]. The strong peak at the corneal surface is an artefact caused by specular reflection that can be observed in a large region of the central part of the cornea. As in this area a large connected and highly correlated structure is present, the IACF is large. It should be noted that the small peak that can be observed in the plots of both subjects at pixel 316 (and pixel 725) is caused by an intensity artefact at this imaging depth that arises from the strong reflection at the corneal surface. For a comparison, the integrated intensity (of each en-face image of the central part of the cornea) was also plotted as function of depth (Figure 6c). In this graph, a slight decrease of the overall intensity with depth can be observed clearly indicating that the signal in Figure 6b is not dominated by varying intensity.

## 4 Discussion

This study demonstrated ultrahigh resolution polarization sensitive OCT with a conical scanning pattern for imaging the human cornea in vivo. Tho improved axial resolution provides a clearer separation between corneal layers and allows for the visualization of very subtle changes, as for example, the thickening of the surface layer at the periphery of the cornea (transition between corneal and limbus epithelium, cf. Figure 2b). A key element for maintaining the high resolution over the entire field of view is the variable dispersion compensatien. As the depth dependent dispersion within the cornea is negligible for our imaging wavelength band, a different and simpler approach was used than what has been introduced quite recently for visible light OCT [23].

In comparison with other ultrahigh resolution OCT systems that have bean presented so far [21,22,24–30], our system provides a large field of view that covers the entire cornea from limbus to limbus. In addition, due to the conical scanning, a relatively high transverse resolution is maintained as there is no need to take care about the trade-off between a sufficiently large depth of focus (for imaging the spherical shaped cornea over the entire field of view) and high transverse resolution. However, in our current configuration, a relatively low sampling density in the slow scanning direction (y-direction) was used in order to keep the measurement time and thus, the motion artifacts low. Visualization of individual cells, such as endothelium cells or nerves in the en-face imaging plane, was not possible with the instrument because of the low sampling density in the y-direction and/or insufficient transverse resolution.

We highlight that the imaging system uses rather low light power (1 mW) on the eye. According to the safety standards and considering that the beam it focused on the cornea, higher light powers may be used. This requires a more powerful light source and/or a different splitting ratio of the fiber beam splitter and may result in a 5–10 dB increased sensitivity. Thus, high quality scans as presented in Figure 3 may be obtained within shorter time and with less image averaging.

Another advantage of the presented system is the additional contrast that is provided by the polarization sensitivity of the instrument. Specifically, the images recorded with the cross polarized OCT channel show different structures (cf. Figure 3) that are not visible in standard OCT images. Previously, this information was used for improved layer segmentation [13]. Thereby, it was assumed that the depolarization of the backscattered light originates only from the stroma. The higher resolution that is provided by the new instrument allows a more precise assessment. As can be seen in Figures 3 and 4, the process of depolarization in the cornea can already be observed within Bowman’s layer. This leads to an observable signal within this layer in the image of the cross-polarization channel (cf. inset of Figure 3b). From a histological point of view, the similar optical behavior (i.e., depolarization) of Bowman’s layer and the stroma seems to be reasonable as Bowman’s layer is regarded as “acellular condensate of the most anterior portion of the stroma” [1]. This finding may be used to investigate an additional parameter that is possibly related to the micro structure of Bowman’s layer and that potentially changes through disease progression, such as Keratoconus.

The information provided by the cross-polarization channel allows the assessment of additional parameters of the corneal stroma. It is known from electron microscopy that the ultra-structure of the corneal stroma changes with depth as deeper layers are more strictly organized than superficial layers [1].

In our en-face images of the cross polarized channel, a gradual change of the image pattern with depth was observed. Starting with very fine structures at the stromal surface, these evolve into larger connected structures towards the posterior surface of the stroma (cf. Figure 5). As the observed pattern shows a similar behavior with depth as the ultrastructure of the stroma, we hypothesized that the images could be used as an indicator for this ultrastructure. Thus, disease related changes of the stromal ultrastructure that currently can only be investigated in vitro might be accessible in vivo via the proposed PS-OCT imaging method.

In order to quantify the depth dependent changes within the stroma, the integrated auto-correlation factor (IACF, cf. Figure 6) was introduced. In principle, the width of the auto correlation function or other metrics for texture analysis might be used as well. The IACFs obtained from the two subjects show a very similar behavior with depth that is independent of the overall signal quality which is quite promising. It should be noted that this behavior could only be observed in both subjects in the central area (4.2 mm diameter) of the stroma as for one subject the signal in the peripheral areas was too low and therefore resulted in erroneous IACF (and thus in a distortion of the curve). The approach in this study may enable the quantitative assessment of the ultrastructure of the stroma. However, further studies with more subjects are needed in order to test the variability of the measured IACF from subject to subject and to investigate potential disease related changes.

Finally, we want to point out that the system is capable of providing the full polarization sensitive information. Thus, the degree of polarization uniformity, retardation and axis orientation of the cornea may be measured similar to our previous work [12].

## 5 Conclusions

Ultrahigh resolution PS-OCT imaging with a conical scanning pattern provides high quality images of the cornea over a large field of view. The variable dispersion compensation is essential for maintaining the high axial resolution across the field of view. The newly introduced IACF may provide a quantitative means for assessing the stromal ultrastructure and corresponding disease related changes.
